# Impact of Working from Home on Cardiovascular Health: An Emerging Issue with the COVID-19 Pandemic

**DOI:** 10.3390/ijerph182211882

**Published:** 2021-11-12

**Authors:** Stefania Angela Di Fusco, Antonella Spinelli, Lorenzo Castello, Edoardo Mocini, Michele Massimo Gulizia, Fabrizio Oliva, Domenico Gabrielli, Giuseppe Imperoli, Furio Colivicchi

**Affiliations:** 1 Clinical and Rehabilitation Cardiology Unit, San Filippo Neri Hospital, Martinotti Street 20, 00135 Rome, Italy; antonella.spinelli@aslroma1.it (A.S.); lorenzo.castello@aslroma1.it (L.C.); furio.colivicchi@aslroma1.it (F.C.); 2 Department of Experimental Medicine, Sapienza University, 00185 Rome, Italy; edoardo.mocini@uniroma1.it; 3 Cardiology Division, Garibaldi-Nesima Hospital, 95122 Catania, Italy; michele.gulizia60@gmail.com; 4 ANMCO Heart Care Foundation, 50121 Florence, Italy; 5 De Gasperis Cardio Center, Niguarda Hospital, 20162 Milano, Italy; fabrizio.oliva@ospedaleniguarda.it; 6 Cardiology/CCU Unit, Cardiovascular Department, San Camillo Hospital, 00152 Rome, Italy; dgabrielli@scamilloforlanini.rm.it; 7 Medicine Unit, Emergency Department, San Filippo Neri Hospital, 00135 Rome, Italy; giuseppe.imperoli@aslroma1.it

**Keywords:** COVID-19 pandemic, working from home, lifestyle, sedentary behavior, physical activity, diet pattern, cardiovascular disease

## Abstract

Mandatory working from home is one of the consequences of the COVID-19 pandemic for a large number of workers. Transition to working from home may significantly impact lifestyle, psychosocial status, and the overall health of workers. This review summarizes available data about the effects of lockdown measures, particularly working from home, on cardiovascular risk factors including sedentary lifestyle, unhealthy diet pattern, psychological distress, smoking, alcohol misuse, and cardiometabolic parameters. Finally, we suggest countermeasures that can attenuate the negative health impact of working from home. Indeed, timely and tailored interventions implemented by companies in cooperation with the health care system could allow workers to benefit more from some of the advantages associated with working from home.

## 1. Introduction

Severe acute respiratory syndrome coronavirus 2 (SARS-CoV-2), which causes coronavirus disease 2019 (COVID-19), emerged in late 2019 and has rapidly evolved into a worldwide pandemic [1]. The COVID-19 pandemic had a wide impact on daily life. This impact is not limited to the direct consequences of viral infection, but also includes the changes in many daily activities such as the way people work. Following the COVID-19 outbreak, many national governments implemented measures to minimize the likelihood that people infected by SARS-CoV-2 would infect others. These lockdown interventions dramatically restricted population mobility and mandated the temporary closure of all non-essential activities and businesses. Many members of the working population were forced to abruptly adapt to profound changes to everyday life. Workers in emergency health settings, supermarket staff, and other essential workers were faced with a significant increase in workload, while office workers able to work remotely were forced to begin working from home, and other employees were out of work due to the shutting down of some businesses.

Working from home refers to work that takes place fully or partially within the worker’s own home, can be performed by both dependent and independent workers, and does not necessarily entail the use of digital devices [2].

This review summarizes available data about the cardiovascular risk factors that were negatively affected by lockdown measures during the COVID-19 pandemic, particularly working from home. We also suggest countermeasures to implement in order to mitigate the negative impact of working from home on cardiovascular health and to benefit more from some of the advantages associated with working from home. Overall, we focus on aspects of disease prevention and health promotion in the emerging setting of working from home. All the possible negative effects of working from home on worker health should be considered when implementing public health policy.

## 2. Literature Search Strategy

**Figure 1:**
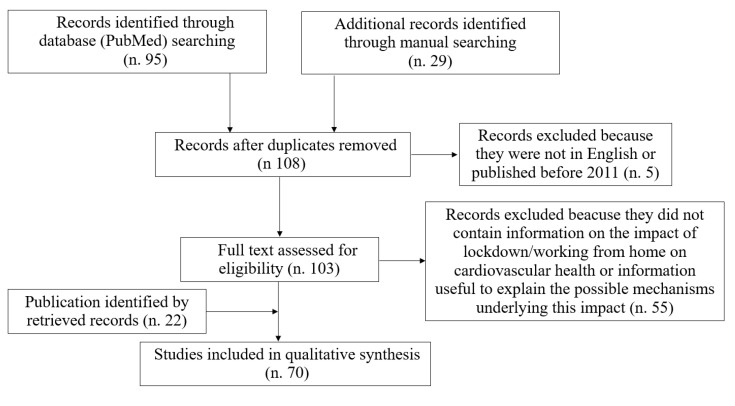
Literature search flowchart.

This narrative review was conceived in light of recent studies that have highlighted that the transition to working from home due to lockdown measures is associated with lifestyle changes and may impact cardiovascular health. In order to assess the risks associated with the behavior changes linked to working from home, we performed a literature search in the PubMed database using the following terms: “working from home” OR “smart working” AND “lifestyle” OR “physical activity” OR “diet” OR “alcohol” OR “smoking” OR “obesity” OR “cardiometabolic disease” OR “health”. Selection criteria were: original articles or review articles published during the last 10 years. References of retrieved records were also manually searched to identify other relevant publications. Articles whose full text was not in English were excluded (Figure 1).

## 3. Scope of the Issue

**Figure 2:**
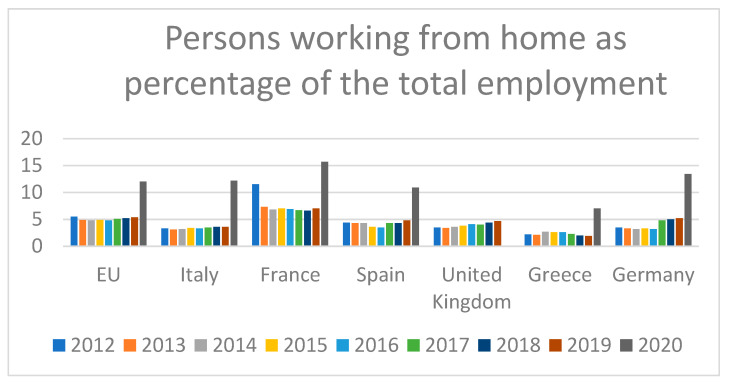
Employees working from home as a percentage of total employment [4].

In Italy in the second quarter of 2020, over four million workers were working from home (19.4% of total workers vs. 4.6% in the second quarter of 2019) [3]. Figure 2 shows the percentages of employees working from home in the European Union (27 countries) and in single countries, including Italy, France, Spain, the United Kingdom, Greece, and Germany, since 2012 [4]. In the United States, it has been reported that the annual percentage of workers working from home at least on an occasional basis increased to 37% in 2015 while the percentage was 9% in 1995 [5]. Of note, the exponential growth in employees working from home is associated with a substantial increase in scientific literature on this topic. Indeed, although the first publication found on PubMed using the search term “working from home” dates back to 1787, until the 1980s, less than 100 publications per year were on this topic [6]. However, scientific interest in working from home has peaked with the COVID-19 pandemic, with 3914 articles on this topic published in 2020 [6].

Even after the end of strict lockdown measures, the governments of several countries encouraged companies to continue remote-working systems for several months to reduce the risk of COVID-19 spread. The change in working conditions, together with the imposed social isolation, resulted in employees staying home longer than before the pandemic and significantly impacted lifestyle, psychosocial status, and the overall health of many individuals worldwide.

### 3.1. Working from Home and Lifestyle Changes

The most obvious consequence of working from home regards lifestyle changes. Working from home is usually associated with long working hours without interruption, which results in lower physical activity and longer sedentary time [7]. In this review, in accordance with the American College of Sports Medicine, “physical activity” refers to any bodily movement that requires energy expenditure above the basal level [8]. During the lockdown period, in addition to the working-from-home mandates for some workers, the prohibition of most outdoor exercise and social activities further contributed to the reduction in physical activity.

A large US survey including 1242 individuals working from home, 288 individuals who lost employment, and 773 individuals without employment change during the COVID-19 pandemic found that both working from home and lost employment were associated with longer time spent in sedentary behavior compared to those who did not change employment status [9]. Working from home was associated with about 31 more minutes per day spent sitting and 33 more minutes of screen time per day [9]. Of note, these changes were independent of prior time spent in sedentary behavior or physical activity [9].

Fukushima et al. conducted an internet-based cross-sectional survey that investigated the association between working from home and physical activity during the COVID-19 pandemic in Japan. This study found significantly shorter time spent in light-intensity physical activity and moderate/vigorous physical activity among those working from home as compared with those not working from home [7]. Furthermore, significantly longer sedentary behavior time and shorter physical activity time were observed among workers with the higher percentage of time spent working from home (76–100%) as compared to subgroups of workers who spent less time working from home (≤75%) [7]. Longer uninterrupted sedentary behavior was reported in all working-from-home groups when compared with the non-working-from-home group [7]. Of note, prolonged sedentary time is associated not only with classical cardio-metabolic risk biomarkers, including waist circumference, high-density lipoprotein cholesterol, triglycerides, and insulin levels, but also with C-reactive protein [10], an inflammatory biomarker associated with an increased risk of several diseases, including cardiovascular disease [11]. Conversely, independent of total time spent in sedentary behavior, interruptions in sedentary behavior are associated with favorable changes in waist circumference, fasting plasma glucose, and C-reactive protein levels [10]. According to these data, sedentary behavior breaks do not necessarily need to include exercise, suggesting that regular breaks could be feasible in several work settings.

Overall, physical inactivity leads to increased cardiovascular disease risk by increasing the burden of established cardiovascular risk factors, including obesity and hypertension [12]. Workers should be counseled regarding the impact of physical activity on health and the risks associated with prolonged sedentary behavior and should be encouraged to initiate and maintain regular physical activity [13]. In order to reduce the deleterious effects of sedentary behavior, interventions aimed at breaking uninterrupted sedentary periods should also be promoted among those working from home. Multiple interventions have been proposed to reduce sitting time in the workplace, such as the use of smartphone applications or other smart devices that monitor sitting time, pedometers, cycling workstations, and height-adjustable workstations [14,15]. Comprehensive approaches should address both environmental constrictions, through facilitating standing and moving more, and organizational barriers, by sharing strategies to move more with colleagues and seniors [14].

Cooperation between health care professionals and workers may help create personalized exercise programs aimed at promoting adherence to healthy behaviors even when working from home. Exercising every day at the same time may be useful to form healthy habits [16]. Further measures that may reduce the negative impact of working from home on lifestyle changes include having a walk outside before starting the workday and having a regular breakfast.

Studies conducted during the COVID-19 pandemic have also reported some changes in eating behaviors. Several surveys reported an increase in snack consumption [17,18,19,20,21] and weight gain [18,19] during lockdown. A large Japanese study including the data of 5929 participants, 27.8% of whom were working from home, reported that those who started working from home during the COVID-19 pandemic increased their intake of vegetables, fruits, dairy products, and snacks and the consumption of self-made meals [20]. This last finding may be due to more free time spent at home, which allowed people to prepare more self-made meals. Female and younger workers seemed to benefit more from the transition to working from home in terms of eating healthier by consuming more self-made meals, vegetables, and fruits. Conversely, in Italy [21] and others western countries [22], a lower consumption of fresh vegetables and fruit during the first lockdown has been reported. However, the working status of the study population was not assessed. The lower consumption of fresh food, which in these studies was associated with “quarantine”, was ascribed to the greater restrictions on mobility imposed in Italy and other western countries during the first lockdown. A further possible cause of the lower consumption of fresh food observed in western countries during lockdown may be due to the more frequent use of home delivery meal services in these countries, which further increased during lockdown.

### 3.2. Working from Home and Psychological Stress

Psychological stress refers to the complex biological and psychological response to environmental demands that exceed individual adaptive capacities [23]. Working from home may pose several psychosocial issues. The absence of in-person interaction with colleagues may generate feelings of isolation and cause anxiety, depression, and sleep disorders [24]. A further stressful aspect of working from home is the absence of clear boundaries between working time and private life, due to the sharing of workspace with family members. This leads to challenges in balancing personal life and leisure activities with work, and often to multitasking. Deterioration in psychological well-being has been reported in some studies conducted during the COVID-19 lockdown [19,25,26], and working from home was found to be a predictor of depressive episodes or increased depressive symptoms [26]. A study that evaluated data from an online survey conducted during the pandemic found family conflict and social isolation to be associated with working-from-home stress [27]. Among 457 individuals who switched to working from home during the COVID-19 pandemic, the reported prevalence of depression, anxiety, and stress was 17.9%, 19.6%, and 19.6%, respectively [28]. In this study, lower levels of physical activity, poor sleep quality, being female, and long working hours were associated with a greater risk of depression among remote workers [28]. Lockdown was also associated with worse sleep quality, with a greater deterioration in sleep quality observed in individuals working from home [29]. A change in sleep schedule was reported among employees of the University of Michigan Medical Center who transitioned to working from home during the pandemic. They more often went to sleep later and woke up later compared to employees who continued to work in person. However, the relevance and clinical impact of this kind of sleep schedule shift needs to be defined [30].

In addition, working from home may result in greater workload with strenuous and longer working hours [31], which may impact overall worker health and increase mortality due to cardiovascular disease [32]. It has been estimated that 3.7% of all deaths from ischemic heart disease and 6.9% of stroke deaths are attributable to working long hours [32]. While working from home, the absence of office distractions may translate into working harder and considerably longer hours than usual, which may lead to a higher cardiovascular disease mortality [32].

Individuals who transitioned to working from home reported poorer sleep quality [33], which is also associated with cardiovascular disease risk [34]. However, in a small sample of office workers in Sweden, working from home during the pandemic was also found to be associated with an increase (34 min) in sleep time [35], with an overall ratio between sleep time and sedentary time during the day a bit closer to Canadian 24-Hour Movement Guideline recommendations [36], implying possible positive health effects. A study that examined the impact of working from home on workers’ mental and physical health found a complex health/work relationship [37]. Differences in company organization and in the support provided by the company seem to be relevant contributors to either increasing or mitigating the negative impact of working from home on mental health [37].

Furthermore, during working from home, psychological stress was more often reported among female workers and workers with lower incomes than among male workers and workers with higher incomes [26]. The difference in the gender impact of working from home on psychological well-being may be due to the greater engagement of women in household activities and childcare, all commitments that add to workload. The association of psychological distress with the income level may be ascribed to the fact that that workers with lower income may have more difficulties to find a dedicated space to work in their home and an adequate workstation. The consequence is a greater difficulty in concentrating on work tasks and even a lower productivity. Living alone has also been found to be a factor that exposes workers to a higher risk of psychological stress due to the greater feel of social isolation while working from home [38]. Of note, a study that evaluated the effects of working from home and working at the office among academic staff found greater parasympathetic activity, assessed by measuring heart rate variability, during working-from-home days [39]. This finding has been attributed to more relaxation when working from home. However, it should be considered that in the population included in this study, working from home was a voluntary choice and was not imposed. In addition, when working-from-home days alternated with working in the office, employees reported lower levels of work–family conflict on working-from-home days [40]. In clinical practice, in order to reduce the negative effects linked to working-from-home stress, it is suggested to set a schedule for daily activities such as establishing what time to wake up, go to sleep, and have meals, and also settling work- and leisure-time hours [41,42].

Furthermore, working from home has also been reported to be associated with increased alcohol and tobacco consumption [43].

Overall, psychosocial distress is known to be associated with an increased risk of cardiovascular disease [44] and may be managed with psycho-educational interventions that include health information, encouraging physical activity implementation by providing appropriate and feasible measures for exercise even at home, emotional/psychosocial support, smoking cessation interventions, and comprehensive alcohol treatment programs [45]. Furthermore, relaxation exercise practice, which seems to play a role in ischemic heart disease secondary prevention [45], can also be included among the interventions aimed at managing psychological distress while working from home. Several findings from experimental and clinical studies strongly indicate the presence of a link between psychological stress associated with work and cardiovascular disease occurrence. It has been estimated that up to 50% increase in cardiovascular disease risk is attributable to a high level of psychological stress due to work [46]. Furthermore, job overload and social isolation, which are often associated with working from home, have been reported to increase the risk of recurrence of cardiovascular disease events and mortality [47]. An Italian study on the effects of smart working on well-being at work suggests that manager and peer support may play a role in workers well-being and may prevent work-life imbalance [48].

In clinical practice, recognizing the conditions that impact the psychological well-being of individuals working from home may allow the implementation of timely psycho-educational interventions, such as optimizing the daily workload and providing psychological support and counseling. Furthermore, when working from home is an employee choice and is alternated with working at the office, it could even have some beneficial effects on the psychological status.

### 3.3. Working from Home and Cardiometabolic Disease

Previously discussed lifestyle changes due to COVID-19-related lockdown and working from home have been found to be strongly associated with weight gain, which has been a common phenomenon during this pandemic and has led to the term “covibesity” being coined [49]. After the first month of lockdown in Italy, a mean self-reported weight gain of about 1.5 kg was reported among 150 adult outpatients with obesity. Of these outpatients, 33% worked from home, 15% still went to the workplace during the lockdown, and 52% did not work. In this study, self-reported anxiety and not consuming healthy foods were significantly associated with greater weight gain [50]. In a survey conducted more than six months after the COVID-19 pandemic outbreak and including 194 employees who switched to working from home because of the pandemic, Guler et al. found that almost half of the study population (46.9%) reported an increase in body weight during the working-from-home period [51]. Another survey, including 869 individuals working from home during the COVID-19 pandemic, reported a 41% prevalence of weight gain, with a dose-response relationship with the intensity of working from home as the number of days per week spent working from home [52]. Of note, a significant association between sedentary work (measured as sitting time) and higher body mass index was already reported in scientific literature [53].

Observational studies have also investigated the impact of lockdown on pre-existing cardiometabolic diseases. A meta-analysis including 10,765 patients with diabetes (1174 with type 1 and 9591 with type 2) reported an improvement in glycemic control during lockdown in type 1 diabetes, and no significant effect on glucose control in type 2 diabetes [54]. Although the information on the share of patients working from home was not available in several of the studies included in this meta-analysis, the increased time available to cope with complex diabetes management, such as glucose monitoring and insulin titration, may at least partially explain the better glycemic control during the lockdown reported in type 1 diabetes patients who stayed home. Of note, the lockdown period as compared with the same period during the previous two years was also associated with a reduction in the number of type 2 diabetes patient visits (on-site or remote), which was significantly greater than the reduction in type 1 diabetes patient visits (−53% vs. −40%; *p* = 0.001) [55]. Furthermore, a telephone survey among type 2 diabetes patients found an increase in carbohydrate and snack consumption and a reduction in glucose self-monitoring during the lockdown in about one quarter of patients, though the rate of working from home in the population studied was not reported [56].

A study that evaluated a population of 72 patients followed at an outpatient neuroendocrine disease clinic showed a significant increase in obesity, dyslipidemia, and metabolic syndrome prevalence at the end of the first lockdown imposed in Italy as compared with the prevalence before the lockdown [57]. However, the prevalence of arterial hypertension, impaired glucose tolerance, and diabetes mellitus did not change. Data on the proportion of patients included in the study who were working from home during the lockdown were not available.

Lockdown measures have been found to be associated with a significant reduction in health care service utilization, which has been found to be reduced by one third compared with pre-pandemic periods [58]. The lower use of health care services may have a negative impact on cardiometabolic disease management by leading to lower physician supervision of chronic treatments and lower patient adherence. Indeed, a trend towards decreased prescription refills for lipid-lowering drugs during lockdown has been reported [59]. The adherence issue observed during lockdown may lead to worse cardiometabolic disease outcomes. Of note, among patients with COVID-19, statin use is a proxy of comorbidities that expose patients to more severe COVID-19 [60], although statin users seem to have a better COVID-19 clinical course than non-statin users [61]. The postulated mechanisms to explain this finding include direct cholesterol-lowering effects on viral infection/replication and several statin pleiotropic effects [62] due to their antithrombotic, anti-inflammatory, and immunomodulatory properties [63]. No specific data are available on the effect of statin use in individuals who developed a “negative” cardiometabolic status, such as “covibesity”, during lockdown. Further research is needed to establish the impact of starting statin treatment for cardiometabolic disease on clinical outcomes in case of COVID-19.

Informing workers of the risks associated with unhealthy behaviors that could be exacerbated by lockdown measures, especially by working from home, may help to reduce the negative effects of these changes on cardiovascular health.

## 4. Discussion

### 4.1. Future Perspectives

Although some lifestyle changes are inevitable in the transition to work from home, greater awareness of the possible negative consequences of this transition can help mitigate these effects. Concerns regarding potential adverse psychosocial effects should not preclude working from home, although individual preventive interventions should be considered [64].

Workers could even take the opportunity of this transition to have a healthier lifestyle. Several strategies to facilitate healthy behavior changes and promote physical activity throughout the workday at home have been proposed and are currently under evaluation. Lockdown measures due to the COVID-19 pandemic provide an opportunity to better understand the impact of working from home on lifestyle and cardiovascular risk.

**Figure 3:**
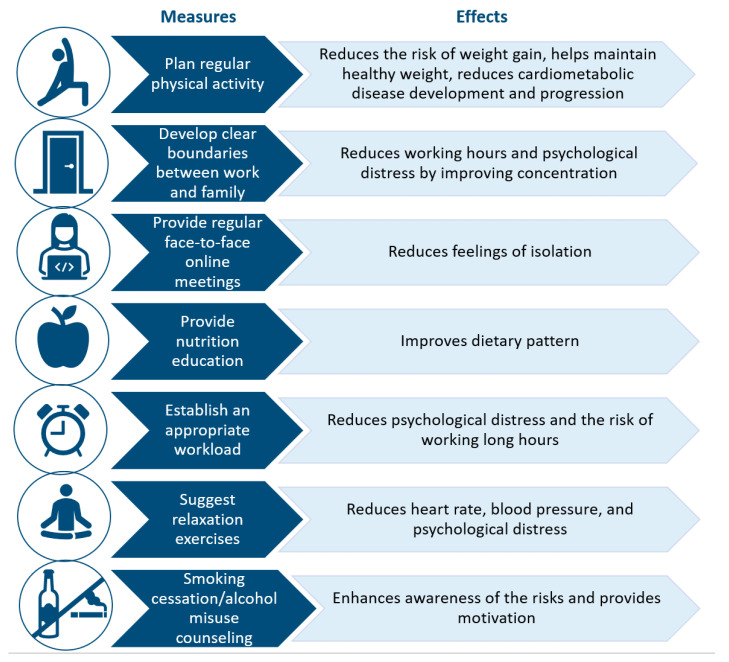
Measures to reduce cardiovascular disease risk while working from home.

Once the negative lifestyle changes associated with working from home that are heightened with lockdown have been identified, it is fundamental to prevent these unhealthy behavior changes in workers transitioning to working from home. It is noteworthy that some positive effects of working from home have been reported, such as increased productivity [31], improved mental well-being due to increased leisure time [38], greater level of flexibility in time management [65] which enables workers to better control and schedule their daily activities, more relaxation [39,65], and the reduced need to commute [24,65]. Furthermore, it should also be taken into account that the impact on mental well-being may depend on the frequency of working from home [66] and on whether it is mandatory or optional. Of note, in a survey study that evaluated the experience of 5748 workers from several different European countries who worked from home during the pandemic, the majority of workers reported a positive experience [67]. However, further research is needed to clarify how to reduce the health risks and increase the benefits of working from home. Interventions aimed at decreasing cardiovascular disease risk that can easily be integrated into the daily life of employees working from home are reported in Figure 3 [68]. It is suggested to address possible psychological and physical issues linked to working from home [69] and to timely implement tailored measures prior to initiating working from home in order to minimize potential negative effects of this transition. Unfortunately, the COVID-19 outbreak was unexpected and has led to mandatory working-from-home transition without adequate preparation for many companies and their workers, which has amplified the occurrence of negative consequences of this transition.

### 4.2. Limitations

In this narrative review, we summarized the main literature data on the impact of working from home on several modifiable cardiovascular risk factors including lifestyle, psycho-social stress, and cardiometabolic parameters. However, some findings are inconsistent across the studies and some limitations of available data must be highlighted. First, although growing scientific literature reports several potential adverse effects associated with working from home, available data comes from observational studies, many of which are online surveys with self-reported outcomes. In addition, there is a huge heterogeneity in the characteristics of individuals included in the different published studies. Furthermore, some of the reported health effects associated with working from home are, at least in part, accentuated by the restriction in population mobility imposed during the COVID-19 pandemic [70]. Indeed, most parts of the studies were conducted during the lockdown. Since the time spent working from home was often a few months, the negative impact of working from home could become even more evident with longer periods of working from home.

## 5. Conclusions

Transition to working from home may have a substantial impact on lifestyle, psychosocial status, and the overall health of workers. In this review, we have summarized available data showing an association between lockdown measures, particularly working from home, and some cardiovascular risk factors. Further studies are needed to establish the impact of working from home lifestyle changes on cardiovascular health and also to identify tailored solutions. Governments, companies, and health care services should cooperate and promote policies to educate workers and advocate healthy lifestyle in the working-from-home setting. Based on available data, we also suggest some countermeasures that may attenuate the possible negative health impact of working from home and could allow workers to benefit more from some of the advantages associated with working from home.

## Abbreviations

The following abbreviations are used in this manuscript:

COVID-19	Coronavirus disease 2019
SARS-CoV-2	Severe acute respiratory syndrome coronavirus 2
